# Cell-Free HPV-DNA as a Biomarker for Oropharyngeal Squamous Cell Carcinoma—A Step Towards Personalized Medicine?

**DOI:** 10.3390/cancers12102997

**Published:** 2020-10-15

**Authors:** Nora Wuerdemann, Rishabh Jain, Anne Adams, Ernst-Jan M. Speel, Steffen Wagner, Simon A. Joosse, Jens P. Klussmann

**Affiliations:** 1 Department of Otorhinolaryngology, Head and Neck Surgery, Medical Faculty, University of Cologne, Kerpener Strasse 62, 50931 Cologne, Germany; s0rijain@uni-bonn.de (R.J.); jens.klussmann@uk-koeln.de (J.P.K.); 2 Center for Molecular Medicine Cologne (CMMC), Medical Faculty, University of Cologne, Robert-Koch-Strasse 21, 50931 Cologne, Germany; 3 Institute of Medical Statistics and Computational Biology, Medical Faculty, University of Cologne, Kerpener Str. 62, 50937 Cologne, Germany; anne.adams@uni-koeln.de; 4 Department of Pathology, GROW-School for Oncology and Developmental Biology, Maastricht University Medical Center, P. Debyelaan 25, 6229 HX Maastricht, The Netherlands; ernstjan.speel@mumc.nl; 5 Department of Otorhinolaryngology, Head and Neck Surgery, University of Giessen, Klinikstrasse 33, 35392 Giessen, Germany; steffen.wagner@hno.med.uni-giessen.de; 6 Department of Tumor Biology, University Medical Center Hamburg-Eppendorf, Martinistrasse 52, 20246 Hamburg, Germany; s.joosse@uke.de; 7 Mildred Scheel Cancer Career Center HaTriCS4, University Medical Center Hamburg-Eppendorf, Martinistrasse 52, 20246 Hamburg, Germany

**Keywords:** oropharyngeal squamous cell carcinoma, human papillomavirus, liquid biopsy, cell-free DNA, biomarker, meta-analysis

## Abstract

**Simple Summary:**

Human papillomavirus (HPV)-related oropharyngeal squamous cell carcinoma (OPSCC) is a distinct tumor entity with relatively favorable overall survival. Nevertheless, up to 25% of HPV-related OPSCC patients develop recurrent or metastatic disease with a fatal outcomes. Biomarkers to enable early diagnosis and to monitor this disease are not established. Liquid biopsy presents a promising minimally invasive method to monitor the cell-free DNA of oncogenic HPV and to enable personalized therapy concepts. Few studies have investigated the role of cell-free HPV DNA (cfHPV-DNA) as a diagnostic marker in patients with OPSCC with variable outcomes. To emphasize the importance of cfHPV-DNA, we performed a literature review and meta-analysis. Our results demonstrate that cfHPV-DNA in patients with OPSCC presents a promising diagnostic tool with high specificity. Nevertheless, further studies with homogeneous inclusion criteria will be necessary to strengthen the role of cfHPV-DNA as a biomarker in the future.

**Abstract:**

Global incidences of oropharyngeal squamous cell carcinoma (OPSCC) are rising due to an association with high-risk human papillomavirus (HPV). Although there is an improved overall survival of HPV-related OPSCC; up to 25% of the patients develop recurrent or distant metastatic disease with a fatal outcomes. Biomarkers to monitor this disease are not established. This meta-analysis reviews the role of cell-free HPV DNA in liquid biopsy (LB) as a biomarker for HPV-related OPSCC. Pubmed, Livivo, and Cochrane Library databases were searched from inception to August, 2020. All studies were analyzed by Meta-DiSc 1.4 and Stata 16.0 statistical software. In total, 16 studies were considered for systematic review, whereas 11 studies met inclusion criteria for meta-analysis, respectively. Pooled sensitivity of cfHPV-DNA at first diagnosis and during follow-up was 0.81 (95% CI; 0.78–0.84) and 0.73 (95% CI; 0.57–0.86), while pooled specificity was 0.98 (95% CI; 0.96–0.99) and 1 (95% CI; 0.99–1). The diagnostic odds ratio (DOR) at first diagnosis was 200.60 (95% CI; 93.31–431.22) and 300.31 (95% CI; 60.94–1479.88) during follow-up. The area under the curve (AUC) of summary receiver operating characteristic (SROC) was 0.99 at first diagnosis and 1.00 during follow-up, respectively. In conclusion, cfHPV-DNA presents a potential biomarker with high specificity in patients with HPV-related OPSCC.

## 1. Introduction

Head and neck squamous cell carcinoma (HNSCC) presents the sixth most common malignancy worldwide and approximately 700,000 new cases are diagnosed each year [1]. Incidences of oropharyngeal squamous cell carcinoma (OPSCC) are rising as a result of an increasing prevalence of high-risk human papillomavirus (HPV)-related tumors and rates have already surpassed those of cervical cancer in some developed countries [2,3,4,5]. Dependent on the geographical region, 40–80% of OPSCC are HPV-related, whereas in other head and neck subsites, rates are estimated below 5% [6]. HPV16 represents the most common high-risk type in OPSCC, with a prevalence of over 90% [3]. It is well established that HPV-related OPSCC comprises a tumor entity with distinct clinical behavior and improved survival rates compared to HPV-negative OPSCC, regardless of the advanced tumor stage [7]. The favorable prognosis has promoted the development of deintensification treatment regimens aiming to spare these patients the devastating side effects of aggressive treatment. Nevertheless, up to 25% of patients develop local/regional recurrent disease or distant metastasis following established therapy concepts and present with 5-year overall survival rates comparable to those observed in HPV-negative OPSCC [8]. This emphasizes that this subgroup of patients would not profit from deintensified treatment regimens [9,10]. Recent methods to monitor OPSCC by clinical screening and imaging are insufficient and new tools that enable early diagnosis, identification of patients with unfavorable outcome, monitoring of treatment response, and detection of minimal residual disease (MRD) are of great importance to guide precision therapy and to improve outcome in these patients.

**Figure 1:**
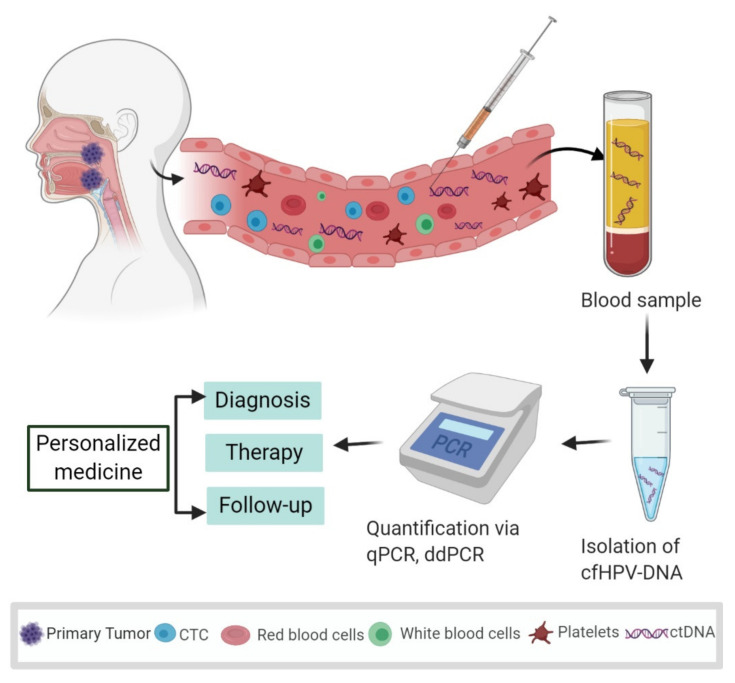
Systematic representation of the process and potential usage of liquid biopsies as a biomarker in oropharyngeal squamous cell carcinoma (OPSCC). Human papillomavirus (HPV) infects the palatine tonsil and the base of the tongue, causing HPV-related OPSCC. Tumor cells and tumor-derived DNA end up in the bloodstream that may function as a biomarker to facilitate personalized medicine. By obtaining blood plasma and isolating the cell-free DNA (cfDNA), quantification methods such as qPCR (quantitative PCR) and droplet digital PCR (ddPCR) make it possible to detect and quantify cell-free HPV-specific DNA (cfHPV-DNA) sequences for diagnosis, therapy stratification, and disease monitoring. Designed with BioRender.com.

Liquid biopsy (LB) analysis is a diagnostic approach to detect, characterize, and monitor the tumor burden using one or more body liquids, including blood, urine, saliva, and spinal fluid. Because tumors may build leaky blood vessels and/or invade the surrounding tissue, their cells and cell products may spill into the blood circulation (Figure 1) [11]. Therefore, blood is the most frequently used source for the isolation of tumor-related markers including circulating tumor cells (CTCs), extracellular vesicles, tumor educated platelets, miRNAs, and circulating tumor DNA (ctDNA) [12]. Detection of ctDNA by identifying specific tumor-related mutations in the blood is considered a highly specific method that was proposed as a biomarker more than two decades ago [13]. The release of tumor DNA into the bloodstream is reported from necrosis or apoptosis of tumor cells [14] or through active secretion of extracellular vesicles [15]. Because the quantity of tumor-derived DNA can be less than 0.1% in the background of normal cell-free DNA (cfDNA), many different and sensitive techniques have been developed for detecting the presence of ctDNA in blood plasma [15]. qPCR (quantitative PCR) or droplet digital PCR (ddPCR) are among the most frequently used techniques due to their ease of use and relatively high sensitivity and specificity for the detection of specific DNA sequences (Figure 1). Owing to the minimal invasiveness of LB, repeated samplings can be performed to monitor dynamic changes of tumor burden. For the detection of minimal residual disease prior to the development of clinical symptoms of recurrence, LB has become a promising approach in breast cancer, prostate cancer, and lung cancer [16,17,18,19]. In those entities, the detection of tumor-specific DNA and respective mutations is in focus to monitor tumor burden and to apply newly developed targeted therapies. On the other hand, monitoring the DNA of an oncogenic virus may be advantageous when tumor growth depends on the activity of the encoded viral oncoproteins. In that case, virus DNA is even a more stable marker than DNA mutations, because it is less dependent on clonal selection processes during tumor progression. HPV-related OPSCC represents such a subgroup of HNSCC that can be specifically monitored due to their virus association by detecting high-risk cell-free HPV DNA (cfHPV-DNA) in the blood, such as has also been shown for Epstein–Barr virus-associated nasopharyngeal cancer [20,21]. Detection of high-risk cfHPV-DNA in LB could provide a future tool for early diagnosis of HPV-related OPSCC. Furthermore, the monitoring of high-risk cfHPV-DNA in sequential LB obtained during systemic therapy or post-surgery might function as a biomarker for the guidance of precision therapy as well as detection of MRD during follow-up. We performed a systematic literature review and meta-analysis to investigate the value of cfHPV-DNA presence in LB as a biomarker in patients with HPV-related OPSCC.

## 2. Results

**Figure 2:**
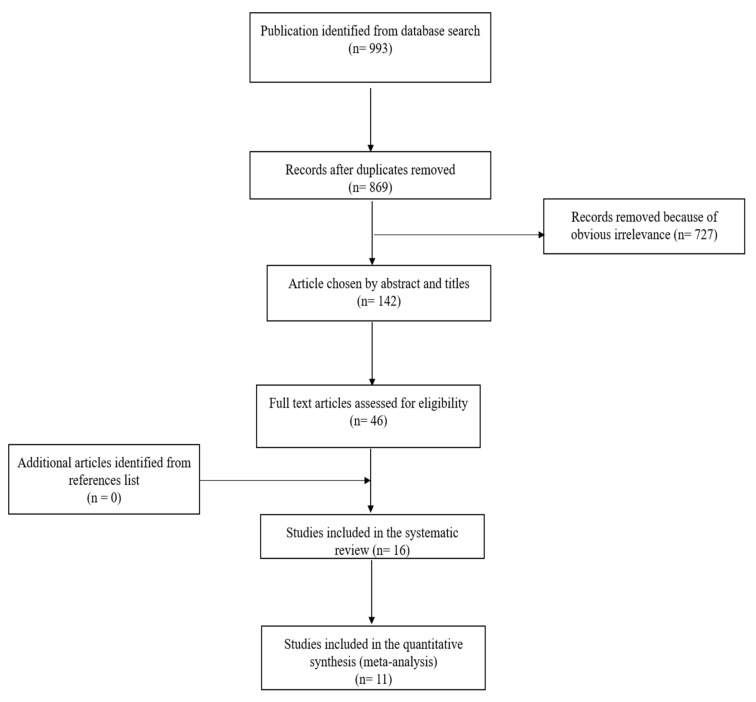
PRISMA (Preferred Reporting Items for Systematic Reviews and Meta-Analysis) flow chart of studies included in systematic review and meta-analysis.

The initial literature search yielded a total of 993 publications (Figure 2). Duplicates (*n* = 124), as well as 727 records, were removed as those articles did not meet the topic of this meta-analysis. One hundred and forty-two articles were selected by abstracts and titles. Subsequently, 46 articles were included for full-text screening. As a result, we obtained 16 records for systematic review (Table 1). Out of these 16, 10 diagnostic studies were eligible for quantitative meta-analysis at first diagnosis of disease (Table 1, marked #), whereas five out of those 16 studies were eligible for analysis of cfHPV-DNA detection during follow-up (Table 1, marked **). In total, data of 998 patients and 286 controls (patients with HPV-negative OPSCC, healthy donors) from 10 studies (USA: six studies; *n* = 696; Europe: three studies; *n* = 279; Australia: one study; *n* = 23) published in 2012–2020 were available. For the performance of the meta-analysis, focusing on first diagnosis, *n* = 594 patients with HPV-related OPSCC and *n* = 286 controls were included. For follow-up analysis, a cohort of *n* = 331 patients with tissue-proven recurrent disease of HPV- related OPSCC and *n* = 84 controls were available. The exclusion of cases according to the initial cohort was due to missing or insufficient HPV status.

### 2.1. Characteristics of Studies Included in Systematic Review and Meta-Analysis

#### 2.1.1. Tumor Characteristics and Treatment

OPSCC patients with UICC (Union for International Cancer Control) stage of 0–IV who received surgery, radio(chemo)therapy, and/or immunotherapy were incorporated (for more information, see Table S1). According to collection of data/samples, five studies had a prospective design [24,27,28,30,37], whereas four studies were purely retrospective [23,25,29,33]. Seven studies did not report how data/samples were collected [22,26,31,32,34,35,36] (Table 1, Table S1).

#### 2.1.2. Samples and Assays Used for Detection of cfHPV-DNA in Blood

In general, polymerase chain reaction (PCR) to quantify circulating cfHPV-DNA was the most common method used. One study [27] used “HPV16-detect” and compared sensitivity and specificity via real-time PCR. Additionally, eight studies [25,28,29,30,33,35,36,37] used droplet digital PCR (ddPCR). Damerla et al. [29] and Jeannot et al. [33] compared ddPCR with qPCR and they reported more sensitive values by using ddPCR at lower cfHPV-DNA concentrations. One study [26] conducted multiplex serologic testing (MST), six studies [22,23,24,29,31,33] used qPCR and two studies also used TaqMan-based telomerase-based reverse transcriptase (TERT) amplification [32,34]. Cao et al. [22] also used conventional PCR. Probes and primers used in the 16 studies included in the review are displayed in Table S3. Thirteen studies extracted cfHPV-DNA from plasma [22,23,25,27,28,29,30,31,32,34,35,36,37], whereas two other studies extracted DNA from serum [24,26] and Jeannot et al. used both plasma and serum [33].

#### 2.1.3. Relationship between cfHPV-DNA Copy Number in Pre-/Post-Treatment Blood Samples and Tumor Stage

The cfHPV-DNA copy number varied considerably among studies at early diagnosis and during treatment. The median plasma cfHPV-DNA copy number was found to be in a range of 222 copies/mL to 880 copies/mL. Two study groups, Dahlstrom et al. [24] and Reder et al. [31], reported the copy number with respect to HPV16 E6 and E7 genes. Kuhs et al. [26] performed multiplex serologic testing and reported that the median fluorescence intensity of the HPV16-E6 gene did not significantly decline over time in post-treatment samples and also did not correlate with the risk of recurrence. Chera et al. [28] proposed a cfHPV-DNA positive clearance profile as having a high baseline copy number (>200 copies/mL) and >95% clearance of cfHPV-DNA by day 28 of chemoradiotherapy. A gradual decline in cfHPV-DNA was seen in patients during chemoradiotherapy throughout the studies. Patients who developed recurrence had an increased level of circulating cfHPV-DNA (542 copies/mL) as observed in Cao et al. [22] (for more information see Table S1).

In the study by Cao et al. [22], a significant correlation of nodal classification and metabolically active nodal volume with HPV-DNA copy number was observed (N1-N2a: 225.5 copies/mL, N2b-N2c: 1026.1 copies/mL, N3: 5500 copies/mL). Five studies [24,31,32,33,36] observed that higher T- and N-stage resulted in higher rates of cfHPV-DNA in pre-treatment samples. In contrast to this, Chera et al. [28] reported that patients with larger tumors may be associated with less release of cfHPV-DNA and, therefore, tumor burden alone may not clarify the variability in the level of pre-treatment cfHPV-DNA in OPSCC patients.

### 2.2. Quality Assessment of Studies Included in Meta-Analysis at First Diagnosis

Quality assessment characteristics of the 10 eligible studies included in the meta-analysis at first diagnosis can be gathered from Supplementary Table S2. The risk of bias was rated high, low, or unclear according to QUADAS-2. Nine studies presented with high quality [22,23,24,25,26,27,28,29,31], whereas one study increased the risk of bias due to an isolated focus on patients with tumor stage III-IV [30].

### 2.3. Diagnostic Accuracy of cfHPV-DNA in OPSCC at First Diagnosis

**Figure 3:**
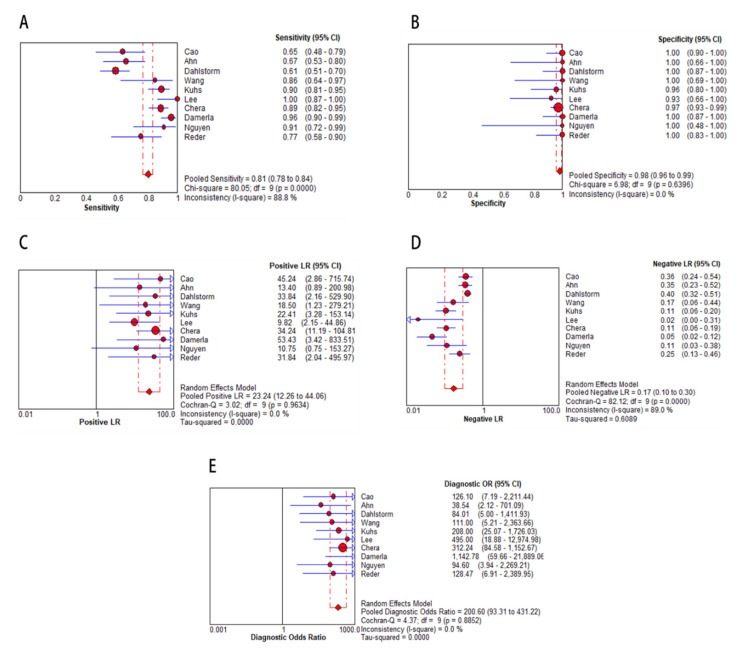
Diagnostic accuracy of cfHPV-DNA displayed by forest plots estimating (**A**) sensitivity, (**B**) specificity, (**C**) positive likelihood ratio (PLR), (**D**) negative likelihood ratio (NLR), and (**E**) diagnostic odds ratio (DOR) at first diagnosis of HPV-related OPSCC (confidence interval (CI) in brackets). The size of red dots represents the sample size included in the studies.

**Figure 4:**
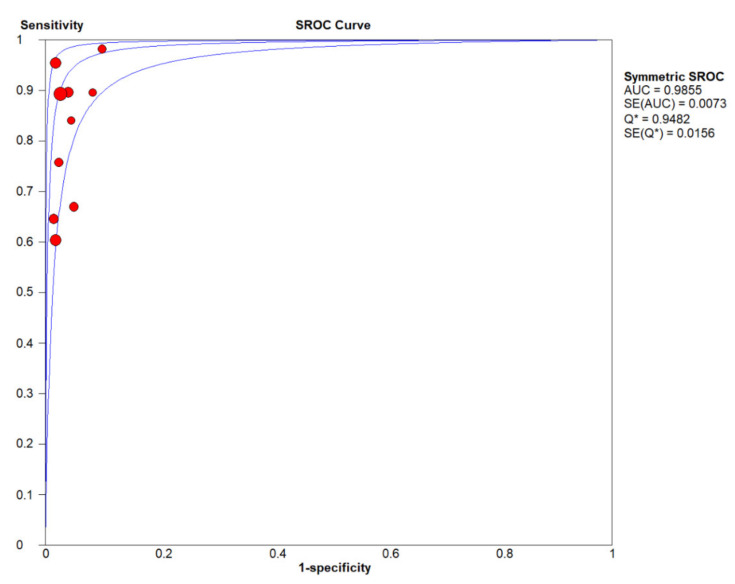
The summary receiver operating characteristic (SROC) curve for analysis of cfHPV-DNA detection at first diagnosis in HPV-related OPSCC. Blue lines represent the SROC curve and standard error (SE). The point where the SROC curve intersects the diagonal and sensitivity equals specificity is defined as Q*. Sensitive versus 1-specificity of each study is plotted as a red dot, whereas size of the dot represents sample size of the study cohort. (# studies included in meta-analysis at first diagnosis)

In the meta-analysis, 10 eligible studies were pooled for diagnostic accuracy of cfHPV-DNA at first diagnosis. The Spearman correlation coefficient was 0.492 (*p* > 0.05, *p* = 0.148), suggesting that the threshold effect was not significant. Therefore, the non-threshold effect was checked by *I^2^* statistics. This analysis demonstrated high heterogeneity in sensitivity (*I^2^* = 88.8%, Figure 3A) with high specificity (no heterogeneity, *I^2^* = 0.0%, Figure 3B). Next, a random effects model was applied to study diagnostic accuracy of circulating cfHPV-DNA in OPSCC. Pooled sensitivity and specificity were 0.81 (95% CI, 0.78–0.84, Figure 3A) and 0.98 (95% CI, 0.96–0.99, Figure 3B), respectively, whereas the pooled positive likelihood ratio (PLR) was 23.24 (95% CI, 12.26-44.06, *I^2^* = 0.0 %, Figure 3C) and the negative likelihood ratio (NLR) was 0.17 (95% CI, 0.10–0.30, *I^2^* = 89.0 %, Figure 3D). Diagnostic odds ratio (DOR) was 200.60 (95% CI, 93.31–431.22, *I^2^* = 0.0 %, Figure 3E). The area under the curve (AUC) of the summary receiver operating characteristic (SROC) was 0.99, indicating a high diagnostic accuracy of cfHPV-DNA in HPV-related OPSCC at first diagnosis (Figure 4).

### 2.4. Subgroup Analysis

To investigate the heterogeneity between the studies, subgroup analysis was performed (Table 2). The following parameters of each study were included: sample source (serum versus plasma), detection method (qPCR vs. ddPCR versus HPV16 detect/multiplex serology testing), patient number (≥ 50 cases versus < 50 cases) and estimation of HPV status based on tissue of the primary (p16 staining versus HPV-PCR versus combined approach). Subgroup analysis based on “sample used” suggested the same specificity of 0.98 with higher sensitivity for plasma (0.85) versus serum (0.73), PLR of 22.79 versus 25.66, NLR of 0.16 versus 0.21, DOR of 216.11 versus 150.09, and AUC of 0.98, respectively. When analyzing “methods used”, ddPCR and HPV16 detect+/MST (multiplex serologic testing) seemed to be more accurate in detecting cfHPV-DNA (sensitivity 0.92, specificity 0.98, PLR 29.29, NLR 0.10, DOR 285.88, AUC 0.97, and sensitivity 0.92, specificity 0.95, PLR 13.49, NLR 0.07, DOR 268.77, AUC n.a.) compared to qPCR (sensitivity 0.65, specificity 1.00, PLR 28.24, NLR 0.37, DOR 85.17, AUC 0.89). According to the “sample size” included in studies, similar estimates with overlapping confidence intervals were detected (sensitivity 0.81, specificity 0.98, PLR 30.19, NLR 0.16, DOR 230.37, AUC 0.99 versus sensitivity 0.82, specificity 0.99, PLR 15.67, NLR 0.21, DOR 148.55, AUC 0.97). Furthermore, subgroup analysis was conducted for different HPV testing methods of the primary tumor, which included p16 staining or HPV-PCR only versus a combined approach (p16 staining, HPV-PCR, in situ hybridization, molecular HPV detection). We found that p16 staining alone and a combined approach demonstrated a higher level of sensitivity and specificity (sensitivity 0.90, specificity 0.98, PLR 28.76, NLR 0.11, DOR 262.75, AUC n.a., and sensitivity 0.85, specificity 0.98, PLR 19.47, NLR 0.16, DOR 197.47, AUC 0.98) than HPV-PCR alone (sensitivity 0.64, specificity 1.00, PLR 24.92, NLR 0.29, DOR 95.49, AUC n.a.).

### 2.5. Publication Bias

**Figure 5:**
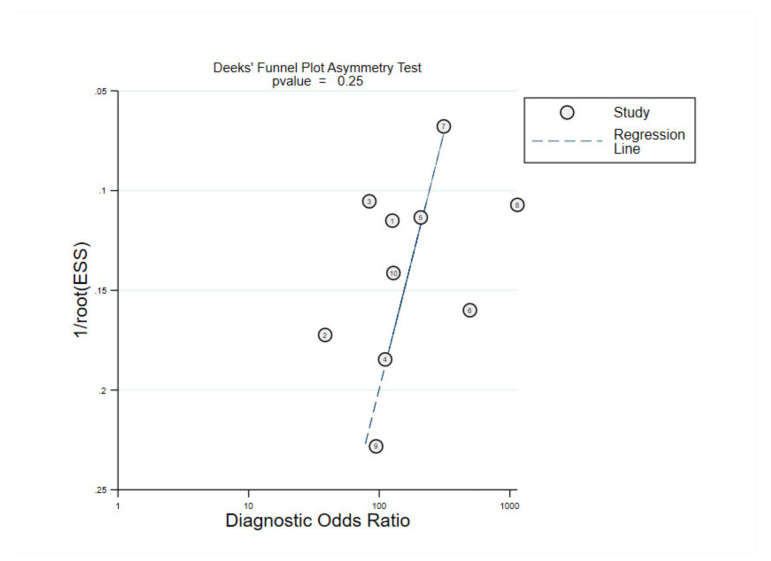
Deeks’ funnel plot for assessment of publication bias.

To estimate publication bias of the 10 included studies in the meta-analysis at first diagnosis, Deeks’ funnel plot asymmetry test was performed (Figure 5). The regression line was associated with a p-value of 0.250, confirming that there was no essential publication bias throughout the included studies.

### 2.6. Diagnostic Accuracy of cfHPV-DNA in OPSCC during Follow-Up

**Figure 6:**
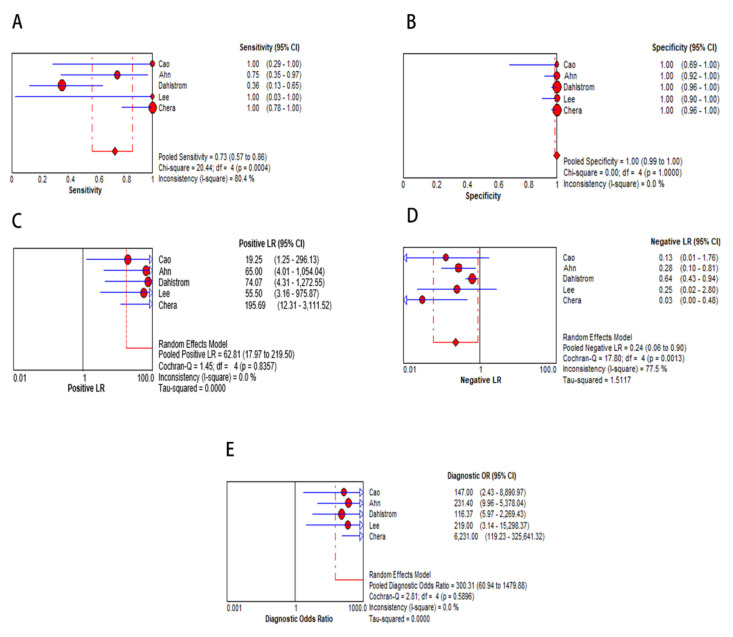
Diagnostic accuracy displayed by forest plots estimating (**A**) sensitivity, (**B**) specificity, (**C**) PLR, (**D**) NLR, and (**E**) DOR for analysis of cfHPV-DNA in detecting recurrence (confidence interval (CI) in brackets). Size of red dots represents sample size included in studies.

**Figure 7:**
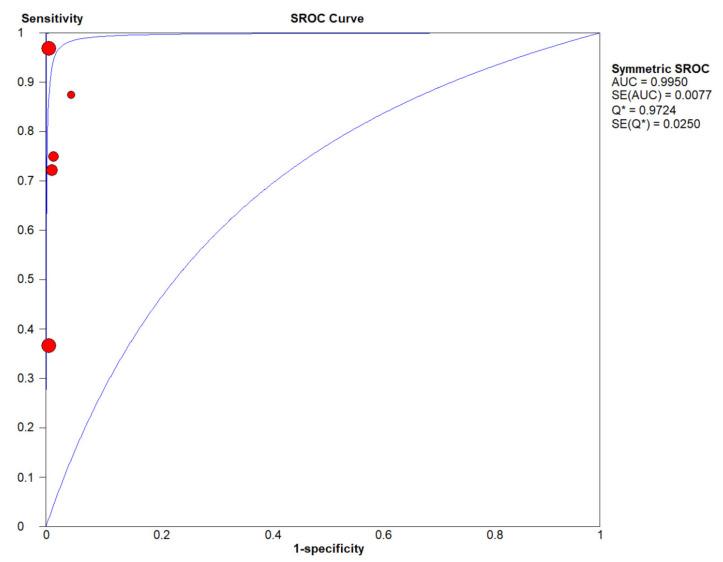
The summary receiver operating characteristic (SROC) curve for detection of cfHPV-DNA as a recurrence marker in HPV-related OPSCC. Blue lines represent the SROC curve and standard error (SE). The point where the SROC curve intersects the diagonal and sensitivity equals specificity is defined as Q*. Sensitive versus 1-specificity of each study is plotted as a red dot, whereas the size of the dot represents the sample size of the study cohort. (** studies included in meta-analysis during follow-up)

In the meta-analysis of follow-up samples, five eligible studies were pooled for diagnostic accuracy as they fulfilled the inclusion criteria as tissue-based confirmation of recurrence and stated true positive (TP), false positive (FP), false negative (FN), and, true negative (TN) diagnostic values (Table 3). The Spearman correlation coefficient was 0.205 (*p* > 0.05, *p* = 0.741), suggesting that the threshold effect was not significant and, therefore, a non-threshold effect was checked by *I^2^* statistics. As this test demonstrated a high heterogeneity in sensitivity (*I^2^* = 80.4%, Figure 6A) and low heterogeneity in specificity (*I^2^* = 0 %, Figure 6B), a random effects model was applied to study diagnostic accuracy of cfHPV-DNA in OPSCC during follow-up. Pooled sensitivity and specificity were 0.73 (95% CI, 0.57–0.86) and one (95% CI, 0.99–1), respectively (Figure 6 A, B). PLR was 62.81 (95% CI, 17.97-219.50, I^2^ = 0 %, Figure 6C), NLR was 0.24 (95% CI, 0.06–0.90, I^2^ = 77.5 %, Figure 6D), and DOR was 300.31 (95% CI, 60.94–1479.88, I^2^ = 0 %, Figure 6E). The AUC of the SROC was 1.0 (Figure 7) with a wide 95% CI, displaying low certainty in the estimate of SROC.

Based on these results, cfHPV-DNA presents a promising biomarker with a high specificity but only moderate sensitivity at first diagnosis and during follow-up in patients with HPV-related OPSCC.

## 3. Discussion

HPV-related OPSCC are often diagnosed in advanced tumor stages due to the development of node metastasis being the first clinical symptoms in many cases. In contrast to HPV-related cervical cancer, precancerous lesions in the oropharynx and reliable screening methods are lacking to date. Regardless of the more favorable prognosis compared to HPV-negative OPSCC, applied treatment regimens do not differ according to virus relation in OPSCC, causing severe side effects in these patients. Risk-adapted, deintensified therapy concepts, currently in the focus of clinical trials, have failed to obtain favorable outcomes by the replacement of chemotherapy by the less toxic EGFR (epidermal growth factor receptor) monoclonal antibody cetuximab [9,10]. Furthermore, up to 25% of patients with HPV-related OPSCC develop recurrent or distant metastatic disease following guideline therapy, resulting in a significantly reduced survival [8]. There is a lack of biomarkers for the early detection of HPV-related cancers at an early stage, for monitoring the disease burden under therapy and during follow-up, and for detecting minimal residual disease. Pathological examination or alternative radiological imaging is the diagnostic gold standard to date. However, dynamic changes of tumor burden during or after treatment cannot be reflected adequately and distant metastases are often not accessible for tissue biopsy, resulting in delayed diagnosis and reduced survival. Monitoring cfHPV-DNA by LB of patients with HPV-related OPSCC could complement conventional imaging and has great potential to improve surveillance by monitoring changes in tumor burden. Recently, this new diagnostic technique has been extensively studied by various research groups. To investigate the diagnostic accuracy of cfHPV-DNA at first diagnosis and during follow-up, we performed a systematic review and meta-analysis focusing on patients with HPV-related OPSCC.

Of 10 individual studies, 549 patients with HPV-related OPSCC were included to investigate the accuracy of cfHPV-DNA at first diagnosis of OPSCC, whereas five studies with 331 patients with HPV-related OPSCC were included in the meta-analysis of follow-up. Pooled sensitivity and specificity were 0.81 (95% CI, 0.78–0.84) and 0.98 (95% CI, 0.96–0.99) at first diagnosis and 0.73 (95% CI, 0.57–0.86) and 1.00 (95% CI, 0.99–1.00) at follow-up, respectively. This demonstrates that the quantification of cfHPV-DNA as a biomarker for HPV-related OPSCC at first diagnosis and at follow-up has high specificity with intermediate sensitivity. Likelihood ratios (LRs) reflect the accuracy of sensitivity and specificity. In this study, the pooled PLR and NLR were 23.24 (95% CI, 12.26–44.06) and 0.17 (95% CI, 0.10–0.30) at first diagnosis and 62.81 (95% CI, 17.97–219.50) and 0.24 (95%, 0.06–0.90) during follow-up, respectively. This result indicated that OPSCC patients have approximately 23% and 63% greater chances of being cfHPV-DNA positive at first diagnosis and during follow-up compared with controls and had approximately 17% and 24% error rates when the true negative was determined in the cfHPV-DNA negative test. In particular, the variety in the sensitivity and NLR reflects poor accuracy of the data. A pooled DOR of 200.60 (95% CI, 93.31–431.22) at first diagnosis and 300.31 (95% CI, 60.94–1479.88) during follow-up indicates a relatively high level of accuracy. Furthermore, the AUC of the SROC curve for the analysis of cfHPV-DNA at first diagnosis was 0.99 and 1.00 during follow-up, suggesting a relatively high accuracy of circulating cfHPV-DNA for OPSCC at first diagnosis in plasma or serum samples. However, during follow-up, there was a wide confidence interval, which reflects low certainty and could be explained by a relatively low number of studies.

In general, our results are in line with a closely related meta-analysis published in 2018 [38]. Jensen et al. also reported high specificity, but lacked the sensitivity of cfHPV-DNA as a biomarker in HPV-associated HNSCC during follow-up. A minor deviation in the results of our meta-analysis at first diagnosis and follow-up might be attributed to a smaller number of studies included in the follow-up analysis. To evaluate the cause of heterogeneity in our study, we additionally performed a quality assessment of included studies and subgroup analysis, which revealed that the method of detection and definition of HPV status (of the primary tumor) were possible factors for heterogeneity. Other factors, such as “specimen used”, might also be responsible, but could not adequately be investigated due to a relatively low number of studies using serum compared to plasma. In addition, the different primers/probes and volumes used throughout the studies might display another source explaining heterogeneity. Nevertheless, due to high variation rates, proper assessment of these factors was not possible. Another fact that has to be taken into account is that detection of cfHPV-DNA is not specific for a certain location of an HPV-related tumor. Although highly unlikely, the positivity of cfHPV-DNA can also occur due to a simultaneous second HPV-related primary other than oropharynx, as cfHPV-DNA is also reported to be a reliable biomarker for cervical carcinoma [39].

Furthermore, it has to be taken into account that no valid data on the correlation of copy number and TNM stage exist to date. Therefore, it might be necessary to increase the volume used for analyses to improve detection rates especially in cases with lower tumor burden. Based on valid correlations, the definition of cut-off values for cfHPV-DNA as a biomarker is inevitable for proper diagnosis and the establishment of clinical guidelines in the future. Additionally, other components obtained from LB, e.g., circulating tumor cells, microRNA, or extracellular vesicles, could complement the detection of cfHPV-DNA as a biomarker and improve predictive accuracy in the future [40,41,42].

LB has numerous potential applications in OPSCC and other cancers. Once validity has been improved, the detection of cfHPV-DNA could function as a screening marker for HPV-associated malignancies at first diagnosis. Furthermore, it can help in planning tailored treatment as well as monitoring therapy response or resistance by sequential use. For example, during follow-up monitoring cfHPV-DNA testing could enable early detection of recurrence and might help to differentiate between tumor activity of an HPV-related OPSCC as distant metastasis or a second primary [43]. This is important especially in the view of de-escalating treatment settings to maintain a sufficient outcome.

As a sensitive marker, LB might help to set accurate time points for radiological imaging, which in turn might minimize radiation exposure. A combination of conventional medical images like PET-CT and blood tests can be safely combined into daily clinical care as both these techniques give insights on the correlation of radiological volume of the tumor with copy numbers of cfHPV-DNA to predict outcome based on two complementary measurements of the tumor burden [44].

Currently, the application of methods such as microarray, next-generation sequencing (NGS) and single-cell RNA sequencing, artificial intelligence, and machine learning algorithms can detect signatures of tumors as mutational changes in multiplexed data [45,46]. Information on viral sequences such as from HPV could be included in these analyses in the future to extend their applicability for the detection of cfHPV DNA in LB.

Limitations of this meta-analysis are the relatively small number of studies included with somewhat low patient and control numbers. Furthermore, we only included studies written in the English language, which could yield selection bias for the language and populations studied. Additionally, there was a high variability throughout study settings and material and methods used for the detection of cfHPV-DNA, which clearly restricts the liability of conclusions drawn. Even though publication bias was not significant and subgroup analysis was performed to evaluate the cause of heterogeneity, the inclusion of only a few factors leaves the risk of not taking other relevant ones into account. Once a higher number of studies is available, a more thorough evaluation of the cause of heterogeneity will be possible to strengthen the role of cfHPV-DNA as a biomarker in HPV-related OPSCC.

## 4. Material and Methods

### 4.1. Search Strategy

This meta-analysis was conducted following the criteria of Preferred Reporting Items for Systematic Reviews and Meta-Analysis (PRISMA) [47]. A systematic literature search was carried out to identify all relevant articles, using Medline (via Pubmed), Cochrane Library databases, and Livivo for the English language. The time period was specified from inception to the 20^th^ of August, 2020. Pubmed Advance Search Builder was used and research articles were selected by using the following terms: “HPV” (human papillomavirus), “head and neck cancer”, “oropharyngeal” OR “oral”, “HPV DNA”, “biomarkers” and “blood” or “serum”, which returned 993 research papers and the following terms were used to get the precise research papers: “oropharyngeal cancer AND HPV cfDNA” OR “oropharynx cancer AND cfDNA” OR “oropharyngeal carcinoma AND cfDNA” OR “circulating DNA AND oropharyngeal cancer”. All searches were first performed by reviewing titles and abstracts by two independent reviewers (NW and RJ) followed by full text screening of the eligible articles. A third reviewer (JPK) was consulted to resolve cases of disagreement. Additionally, reference lists of the included studies were assessed and screened to extract relevant information on the related topics.

### 4.2. Inclusion and Exclusion Criteria

In the meta-analysis, only those studies were included if they met the following criteria: (a) estimating cfHPV-DNA in the blood of patients with OPSCC; (b) including a minimum of 10 HPV-related OPSCC patients along with negative controls; (c) methods for the detection of viral DNA and target genes clearly defined; (d) studies published in the English language; (e) diagnostic value of cfHPV-DNA in OPSCC was stated or could be calculated at first diagnosis; (f) for meta-analysis of follow-up, stated tissue-based confirmation of the recurrence of HPV-related OPSCC and the diagnostic value of HPV blood and biopsy relapse had to be stated additionally and clearly defined or able to be calculated during follow-up. 

The following characteristics were excluded: (a) diagnostic values could not be retrieved from incomplete data to construct a 2 × 2 table; (b) sample size < 10; (c) records available in languages other than English; (d) repeated studies that overlapped included studies; (e) experiments based on cell lines; (f) circulating tumor cells or other genetic markers.

### 4.3. Data Extraction

The following data were collected from each of the eligible studies: last name of the first author, year, country, study design, tumor location, demographic variables, method for cfHPV-DNA assessment including cfDNA extraction methods and collection, tumor stage, sample type, and diagnostic performance including detection rate, true positive (TP), false positive (FP), true negative (TN), false negative (FN), specificity, and sensitivity. For the follow-up, “Tissue +, Blood +” (TP), “Tissue −, Blood +” (FP), “Tissue +, Blood −” (FN), and “Tissue −, Blood −” (TN) relapse were extracted. Furthermore, the correlation of cfHPV-DNA presence with tumor and nodal status along with the positive trend of pre- and post-therapeutic cfHPV-DNA detection were extracted.

### 4.4. Quality Assessment

To evaluate the potential risk of bias and quality of included studies, QUADAS-2 guidelines were used for evaluation. The risk of bias was rated high (H), low (L), or unclear (U) according to QUADAS-2 [48].

### 4.5. Statistical Analysis

We used standard methods suggested for meta-analysis of diagnostic test evaluations [47]. Meta-analysis was performed by using Meta-DiSc 1.4 (Cochrane Colloquium, Barcelona, Spain) and Stata 16.0 (Stata Corporation, College Station, TX, USA) statistical software. Indicators like sensitivity, specificity, diagnostic odds ratio (DOR), positive likelihood ratio (PLR), and negative likelihood ratio (NLR) were summarized in bivariate meta-analysis model and displayed by standard forest plot. By plotting the sensitivity and specificity of each of the studies, bivariate SROCs with a 95% confidence interval (95% CI) were produced. The area under the curve (AUC) of the SROC was used to predict overall accuracy. The threshold effect was detected by the Spearman correlation coefficient and values of *p* < 0.05 which indicated significant threshold effects. Heterogeneity between studies was evaluated through chi-squared and I^2^ tests. Values of *p* < 0.1 or I^2^ higher than 50% indicated the existence of significant heterogeneity [39]. Subgroup analysis was performed to explore the possibility of heterogeneity [49], whereas publication bias was assessed by Deeks’ funnel plot asymmetry test [50].

## 5. Conclusions

In conclusion, cfHPV-DNA in the blood of patients with HPV-related OPSCC presents a potential biomarker with high specificity at first diagnosis and during follow-up. Testing for cfHPV-DNA proved to be a promising application of liquid biopsy for early detection of primary OPSCC in high-risk groups such as immunodeficient patients. Heterogeneity of sensitivity and NLR could be explained by different specimens and methods used for the detection of cfHPV-DNA, as well as variability in the estimation of HPV status in the primary. Although publication bias was ruled out, our analyses suggest that additional studies with larger sample sizes and homogeneous study protocols are necessary in the future to increase sensitivity and to further investigate proof diagnostic accuracy in patients with HPV-related OPSCC.

## Figures and Tables

**Table 1:** Summary of all 16 studies assessing blood cfHPV-DNA in HPV-related OPSCC included in review and meta-analysis.

Study	Year	Country	Study Design	Patients (Total/ Control) (*n*)	Primary Site of Tumor (*n*)	Tissue HPV+ (*n*)	Method/Assay	Tumor Stage	Sample	TP	FP	FN	TN	Sensitivity (%)	Specificity (%)
1. Cao et al. [22] #**	2012	USA	NR	40/34	OPSCC (40)	40	cPCR qPCR	I-IV	Plasma	26	0	14	34	65%	100%
2. Ahn et al. [23] #**	2014	USA	Retrospective	93/9	OPSCC (87) US (6)	52	qPCR	0-IV	Plasma	35	0	17	9	67%	100%
3. Dahlstrom et al. [24] #**	2015	USA	Prospective	262/27	OPSCC (262)	114	qPCR	I-IV	Serum	69	0	45	27	61%	100%
4. Wang et al. [25] #	2015	USA	Retrospective	93/10	OC (46) OPSCC (34) L(10) HP(3)	21	ddPCR	I-IV	Plasma	18	0	3	10	86%	100%
5. Kuhs et al. [26] #	2017	Germany	NR	161/25	OPSCC (87)	87	Multiplex serologic testing	I-IV	Serum	78	1	9	24	89.70%	96%
6. Lee et al. [27] #**	2017	England	Prospective	88/14 Test: 55 Validation: 33	Test cohort OPSCC (47) L (4) HP (4)	27 (Test cohort)	HPV16-detect	I-IV	Plasma	27	1	0	13	100%	93%
7. Chera et al. [28] #	2019	USA	Prospective	103/115	103 (OPSCC)	103	ddPCR	0-IV	Plasma	92	3	11	112	89%	97%
8. Damerla et al. [29] #	2019	USA	Retrospective	105/27	97 (OPSCC) 8 (ASCC)	97	ddPCR qPCR	0-IV	Plasma	93	0	4	27	96%	100%
9. Nguyen et al. [30] #	2020	Australia	Prospective	23/5	OPSCC (23)	23	ddPCR	III-IV	Plasma	21	0	2	5	91%	100%
10. Reder et al. [31] #	2020	Germany	NR	30/20	OPSCC (30)	30	qPCR	I-IV	Plasma	23	0	7	20	76.60%	100%
Study	Year	Country	Study design	Patients (Total/ Control) (*n*)	Primary site of tumor (*n*)	Tissue HPV+ (*n*)	Method/ Assay	Tumor stage	Sample	TP	FP	FN	TN	Sensitivity (%)	Specificity (%)
11. Mazurek et al. [32]	2016	Poland	NR	200/15	72 (OPSCC)	NR	TaqMan-based TERT amplification	I-IV	Plasma	NR	NR	NR	NR	n.a.	n.a.
12. Jeannot et al. [33]	2016	France	Retrospective	70/18	UC (47) ASCC (15) HNSCC (8)	NR	ddPCR qPCR	II, IV	Serum/Plasma	8	0	0	18	100%	100%
13. Rutkowski et al. [34]	2017	Poland	NR	179/NR	OPSCC (55)	47	TaqMan-based TERT amplification	I-IV	Plasma	NR	NR	NR	NR	n.a.	n.a.
14. Hanna et al. [35]	2018	USA	NR	22/NR	OPSCC (22)	22	ddPCR	I-IV	Plasma	NR	NR	NR	NR	n.a.	n.a.
15. Veyer et al. [36]	2019	France	NR	66/NR	OPSCC (66)	66	ddPCR	I-IV	Plasma	47	NR	19	NR	71%	n.a.
16. Chera et al. [37] **	2020	USA	Prospective	115/NR	OPSCC (115)	115	ddPCR	I-III	Plasma	NR	NR	NR	NR	n.a.	n.a.

**Abbreviations**: NR—Not reported, ddPCR—Droplet digital PCR, qPCR— Quantitative PCR, cPCR—Conventional PCR, TP—True positive, FP—False positive, FN—False negative, TN—True negative, OPSCC—Oropharyngeal squamous cell carcinoma, US—Unknown site, OC—Oral cavity, L—Larynx, HP—Hypopharynx, ASCC—Anal squamous cell carcinoma, UC—Uterine cervix, HNSCC—Head and neck squamous cell carcinoma, n.a.—not applicable. # studies included in meta-analysis at first diagnosis and ** studies included in meta-analysis during follow-up.

**Table 2:** Results of the subgroup analysis.

Subgroup	No. of Studies	Sensitivity (95% CI)	Specificity (95% CI)	PLR (95% CI)	NLR (95% CI)	DOR (95% CI)	AUC
Overall	10	0.81 (0.78–0.84)	0.98 (0.96–0.99)	23.24 (12.26–44.06)	0.17 (0.10–0.30)	200.60 (93.31–431.22)	0.99
Sample							
Plasma	8	0.85 (0.81–0.89)	0.98 (0.96–1.00)	22.79 (11.32–45.89)	0.16 (0.09–0.29)	216.11 (91.63–509.69)	0.98
Serum	2	0.73 (0.66–0.79)	0.98 (0.90–1.00)	25.66 (5.31–123.99)	0.21 (0.05–0.95)	150.09 (27.61–815.77)	n.a.
Method							
qPCR	4	0.65 (0.58–0.71)	1.00 (0.96–1.00)	28.24 (7.17–111.20)	0.37 (0.31–0.44)	85.17 (20.21–358.89)	0.89
ddPCR	4	0.92 (0.88–0.95)	0.98 (0.95–1.00)	29.29 (11.80–72.74)	0.10 (0.06–0.16)	285.88 (100.03–817.00)	0.97
HPV16-detect+/ MST	2	0.92 (0.86–0.96)	0.95 (0.83–0.99)	13.49 (4.10–44.41)	0.07 (0.01–0.38)	268.77 (45.51–1587.28)	n.a.
Sample Size							
Greater than 50	5	0.81 (0.77–0.85)	0.98 (0.95–0.99)	30.19 (13.23–68.85)	0.16 (0.06–0.40)	230.37 (91.35–580.98)	0.99
Less than 50	5	0.82 (0.74–0.88)	0.99 (0.93–1.00)	15.67 (5.69–43.14)	0.21 (0.11–0.38)	148.55 (38.03–580.31)	0.97
HPV Tissue Status							
p16 staining	2	0.90 (0.83–0.94)	0.98 (0.93–0.99)	28.76 (10.26–80.64)	0.11 (0.07–0.18)	262.75 (78.51–879.35)	n.a.
HPV-PCR	2	0.64 (0.56–0.72)	1.00 (0.91–1.00)	24.92 (3.61–172.05)	0.29 (0.11–0.74)	95.49 (12.00–759.73)	n.a.
Combined approach	6	0.85 (0.80–0.88)	0.98 (0.95–1.00)	19.47 (7.92–47.86)	0.16 (0.08–0.33)	197.47 (64.08–608.52)	0.98

**Abbreviations:** n.a.—not applicable; AUC—Area under the curve (cannot be calculated if there are only two studies in the subgroup), PLR—Positive likelihood ratio, NLR—Negative likelihood ratio, DOR—Diagnostic odds ratio, CI—Confidence interval, ddPCR—Droplet digital PCR, qPCR—Quantitative PCR.

**Table 3:** Studies included in the meta-analysis of follow-up stating relapse of HPV-related OPSCC according to tissue and blood sample testing.

Study	Median Follow-up	Year	Tissue + Blood + (TP)	Tissue − Blood + (FP)	Tissue + Blood − (FN)	Tissue − Blood − (TN)
Cao et al. [22] **	12-22 months	2012	3	0	0	10
Ahn et al. [23]	49 months	2014	6	0	2	44
Dahlstrom et al. [24]	67 months	2015	5	0	9	100
Lee et al. [27]	12 weeks	2017	1	0	0	36
Chera et al. [37]	23 months	2019	15	0	0	100

** Follow-up plasma was not available for one patient with recurrent HPV-related OPSCC. Abbreviations: TP—True positive, FP—False positive, FN—False negative, TN—True negative.

## Supplementary Materials

The following are available online at https://www.mdpi.com/2072-6694/12/10/2997/s1. Table S1: Extra information on studies included in the review and meta-analysis. Table S2: Quality assessment of the studies included in meta-analysis. Table S3: Primers and probes used in the different studies of the review and meta-analysis.

## References

[1] Ferlay J., Colombet M., Soerjomataram I., Mathers C., Parkin D.M., Piñeros M., Znaor A., Bray F. (2019). Estimating the Global Cancer Incidence and Mortality in 2018: GLOBOCAN Sources and Methods. Int. J. Cancer.

[2] CDC A-Z Index. https://www.cdc.gov/az/h.html.

[3] Wittekindt C., Wagner S., Bushnak A., Prigge E.-S., Von Doeberitz M.K., Würdemann N., Bernhardt K., Pons-Kühnemann J., Maulbecker-Armstrong C., Klussmann J.P. (2019). Increasing Incidence Rates of Oropharyngeal Squamous Cell Carcinoma in Germany and Significance of Disease Burden Attributed to Human Papillomavirus. Cancer Prev. Res..

[4] De Martel C., Plummer M., Vignat J., Franceschi S. (2017). Worldwide Burden of Cancer Attributable to HPV by Site, Country and HPV Type. Int. J. Cancer.

[5] Chaturvedi A.K., Engels E.A., Pfeiffer R.M., Hernandez B.Y., Xiao W., Kim E., Jiang B., Goodman M.T., Sibug-Saber M., Cozen W. (2011). Human Papillomavirus and Rising Oropharyngeal Cancer Incidence in the United States. J. Clin. Oncol..

[6] Boscolo-Rizzo P., Del Mistro A., Bussu F., Lupato V., Baboci L., Almadori G., Da Mosto M.C., Paludetti G. (2013). New Insights into Human Papillomavirus-Associated Head and Neck Squamous Cell Carcinoma. Acta Otorhinolaryngol. Ital..

[7] Klussmann J.P., Gültekin E., Weissenborn S.J., Wieland U., Dries V., Dienes H.P., Eckel H.E., Pfister H.J., Fuchs P.G. (2003). Expression of P16 Protein Identifies a Distinct Entity of Tonsillar Carcinomas Associated with Human Papillomavirus. Am. J. Pathol..

[8] Faraji F., Eisele D.W., Fakhry C. (2017). Emerging Insights into Recurrent and Metastatic Human Papillomavirus-Related Oropharyngeal Squamous Cell Carcinoma. Laryngosc. Investig. Otolaryngol..

[9] Mehanna H., Robinson M., Hartley A., Kong A., Foran B., Fulton-Lieuw T., Dalby M., Mistry P., Sen M., O’Toole L. (2019). Radiotherapy plus Cisplatin or Cetuximab in Low-Risk Human Papillomavirus-Positive Oropharyngeal Cancer (De-ESCALaTE HPV): An Open-Label Randomised Controlled Phase 3 Trial. Lancet.

[10] Gillison M.L., Trotti A.M., Harris J., Eisbruch A., Harari P.M., Adelstein D.J., Jordan R.C.K., Zhao W., Sturgis E.M., Burtness B. (2019). Radiotherapy plus Cetuximab or Cisplatin in Human Papillomavirus-Positive Oropharyngeal Cancer (NRG Oncology RTOG 1016): A Randomised, Multicentre, Non-Inferiority Trial. Lancet.

[11] Joosse S.A., Gorges T.M., Pantel K. (2015). Biology, Detection, and Clinical Implications of Circulating Tumor Cells. EMBO Mol. Med..

[12] Joosse S.A., Pantel K. (2015). Tumor-Educated Platelets as Liquid Biopsy in Cancer Patients. Cancer Cell.

[13] Sidransky D. (1997). Nucleic Acid-Based Methods for the Detection of Cancer. Science.

[14] Kunnath A.P., Priyashini T. (2019). Potential Applications of Circulating Tumor DNA Technology as a Cancer Diagnostic Tool. Cureus.

[15] Elazezy M., Joosse S.A. (2018). Techniques of Using Circulating Tumor DNA as a Liquid Biopsy Component in Cancer Management. Comput. Struct. Biotechnol. J..

[16] Bidard F.-C., Michiels S., Riethdorf S., Mueller V., Esserman L.J., Lucci A., Naume B., Horiguchi J., Gisbert-Criado R., Sleijfer S. (2018). Circulating Tumor Cells in Breast Cancer Patients Treated by Neoadjuvant Chemotherapy: A Meta-Analysis. J. Natl. Cancer Inst..

[17] Chung C., Ma H. (2017). Driving Toward Precision Medicine for Acute Leukemias: Are We There Yet?. Pharmacother. J. Hum. Pharmacol. Drug Ther..

[18] Lorente D., Olmos D., Mateo J., Bianchini D., Seed G., Fleisher M., Danila D.C., Flohr P., Crespo M., Figueiredo I. (2016). Decline in Circulating Tumor Cell Count and Treatment Outcome in Advanced Prostate Cancer. Eur. Urol..

[19] Krebs M.G., Sloane R., Priest L., Lancashire L., Hou J.-M., Greystoke A., Ward T.H., Ferraldeschi R., Hughes A., Clack G. (2011). Evaluation and Prognostic Significance of Circulating Tumor Cells in Patients With Non–Small-Cell Lung Cancer. J. Clin. Oncol..

[20] Wang W.-Y., Twu C.-W., Chen H.-H., Jiang R.-S., Wu C.-T., Liang K.-L., Shih Y.-T., Chen C.-C., Lin P.-J., Liu Y.-C. (2013). Long-Term Survival Analysis of Nasopharyngeal Carcinoma by Plasma Epstein-Barr Virus DNA Levels. Cancer.

[21] Wang W.-Y., Lin T.-Y., Twu C.-W., Tsou H.-H., Lin P.-J., Liu Y.-C., Huang J.-W., Hsieh H.-Y., Lin J.-C. (2016). Long-Term Clinical Outcome in Nasopharyngeal Carcinoma Patients with Post-Radiation Persistently Detectable Plasma EBV DNA. Oncotarget.

[22] Cao H., Banh A., Kwok S., Shi X., Wu S., Krakow T., Khong B., Bavan B., Bala R., Pinsky B.A. (2012). Quantitation of Human Papillomavirus DNA in Plasma of Oropharyngeal Carcinoma Patients. Int. J. Radiat. Oncol. Biol. Phys..

[23] Ahn S.M., Chan J.Y.K., Zhang Z., Wang H., Khan Z., Bishop J.A., Westra W., Koch W.M., Califano J.A. (2014). Saliva and Plasma Quantitative Polymerase Chain Reaction-Based Detection and Surveillance of Human Papillomavirus-Related Head and Neck Cancer. JAMA Otolaryngol. Head Neck Surg..

[24] Dahlstrom K.R., Li G., Hussey C.S., Vo J.T., Wei Q., Zhao C., Sturgis E.M. (2015). Circulating Human Papillomavirus DNA as a Marker for Disease Extent and Recurrence among Patients with Oropharyngeal Cancer. Cancer.

[25] Wang Y., Springer S., Mulvey C.L., Silliman N., Schaefer J., Sausen M., James N., Rettig E.M., Guo T., Pickering C.R. (2015). Detection of Somatic Mutations and HPV in the Saliva and Plasma of Patients with Head and Neck Squamous Cell Carcinomas. Sci. Transl. Med..

[26] Kuhs K.A.L., Kreimer A.R., Trivedi S., Holzinger D., Pawlita M., Pfeiffer R.M., Gibson S.P., Schmitt N.C., Hildesheim A., Waterboer T. (2017). Human Papillomavirus 16 E6 Antibodies Are Sensitive for Human Papillomavirus–Driven Oropharyngeal Cancer and Are Associated with Recurrence. Cancer.

[27] Lee J.Y., Garcia-Murillas I., Cutts R.J., De Castro D.G., Grove L., Hurley T., Wang F., Nutting C., Newbold K., Harrington K. (2017). Predicting Response to Radical (Chemo)Radiotherapy with Circulating HPV DNA in Locally Advanced Head and Neck Squamous Carcinoma. Br. J. Cancer.

[28] Chera B.S., Kumar S., Beaty B.T., Marron D., Jefferys S., Green R., Goldman E.C., Amdur R., Sheets N., Dagan R. (2019). Rapid Clearance Profile of Plasma Circulating Tumor HPV Type 16 DNA during Chemoradiotherapy Correlates with Disease Control in HPV-Associated Oropharyngeal Cancer. Clin. Cancer Res..

[29] Damerla R.R., Lee N.Y., You D., Soni R., Shah R., Reyngold M., Katabi N., Wu V., McBride S.M., Tsai C.J. (2019). Detection of Early Human Papillomavirus–Associated Cancers by Liquid Biopsy. JCO Precis. Oncol..

[30] Nguyen B., Meehan K., Pereira M.R., Mirzai B., Lim S.H., Leslie C., Clark M., Sader C., Friedland P., Lindsay A. (2020). A Comparative Study of Extracellular Vesicle-Associated and Cell-Free DNA and RNA for HPV Detection in Oropharyngeal Squamous Cell Carcinoma. Sci. Rep..

[31] Reder H., Taferner V.F., Wittekindt C., Bräuninger A., Speel E.-J.M., Gattenlöhner S., Wolf G., Klussmann J.P., Wuerdemann N., Wagner S. (2020). Plasma Cell-Free Human Papillomavirus Oncogene E6- and E7-DNA Predicts Outcome in Oropharyngeal Squamous Cell Carcinoma. J. Mol. Diagn..

[32] Mazurek A.M., Rutkowski T., Fiszer-Kierzkowska A., Małusecka E., Składowski K. (2016). Assessment of the Total CfDNA and HPV16/18 Detection in Plasma Samples of Head and Neck Squamous Cell Carcinoma Patients. Oral. Oncol..

[33] Jeannot E., Becette V., Campitelli M., Calméjane M.-A., Lappartient E., Ruff E., Saada S., Holmes A., Bellet D., Sastre-Garau X. (2016). Circulating Human Papillomavirus DNA Detected Using Droplet Digital PCR in the Serum of Patients Diagnosed with Early Stage Human Papillomavirus-Associated Invasive Carcinoma. J. Pathol. Clin. Res..

[34] Rutkowski T., Mazurek A., Snietura M. (2017). Post-Treatment Circulating Free HPV DNA As a Marker of Treatment Outcome in Patients with HPV-Related Propharyngeal Cancer After Radio(Chemo)Therapy. Cell. Mol. Med. Open Access.

[35] Hanna G.J., Supplee J.G., Kuang Y., Mahmood U., Lau C.J., Haddad R.I., Jänne P.A., Paweletz C.P. (2018). Plasma HPV Cell-Free DNA Monitoring in Advanced HPV-Associated Oropharyngeal Cancer. Ann. Oncol..

[36] Veyer D., Wack M., Mandavit M., Garrigou S., Hans S., Bonfils P., Tartour E., Bélec L., Wang-Renault S.-F., Laurent-Puig P. (2020). HPV Circulating Tumoral DNA Quantification by Droplet-Based Digital PCR: A Promising Predictive and Prognostic Biomarker for HPV-Associated Oropharyngeal Cancers. Int. J. Cancer.

[37] Chera B.S., Kumar S., Shen C., Amdur R., Dagan R., Green R., Goldman E., Weiss J., Grilley-Olson J., Patel S. (2020). Plasma Circulating Tumor HPV DNA for the Surveillance of Cancer Recurrence in HPV-Associated Oropharyngeal Cancer. J. Clin. Oncol..

[38] Jensen K.K., Grønhøj C., Jensen D.H., von Buchwald C. (2018). Circulating Human Papillomavirus DNA as a Surveillance Tool in Head and Neck Squamous Cell Carcinoma: A Systematic Review and Meta-Analysis. Clin. Otolaryngol..

[39] Gu Y., Wan C., Qiu J., Cui Y., Jiang T., Zhuang Z. (2020). Circulating HPV CDNA in the Blood as a Reliable Biomarker for Cervical Cancer: A Meta-Analysis. PLoS ONE.

[40] Peacock B., Rigby A., Bradford J., Pink R., Hunter K., Lambert D., Hunt S. (2018). Extracellular Vesicle MicroRNA Cargo Is Correlated with HPV Status in Oropharyngeal Carcinoma. J. Oral Pathol. Med..

[41] Hess A.-K., Müer A., Mairinger F.D., Weichert W., Stenzinger A., Hummel M., Budach V., Tinhofer I. (2017). MiR-200b and MiR-155 as Predictive Biomarkers for the Efficacy of Chemoradiation in Locally Advanced Head and Neck Squamous Cell Carcinoma. Eur. J. Cancer.

[42] Hofmann L., Ludwig S., Vahl J.M., Brunner C., Hoffmann T.K., Theodoraki M.-N. (2020). The Emerging Role of Exosomes in Diagnosis, Prognosis, and Therapy in Head and Neck Cancer. Int. J. Mol. Sci..

[43] Spector M.E., Farlow J.L., Haring C.T., Brenner J.C., Birkeland A.C. (2018). The Potential for Liquid Biopsies in Head and Neck Cancer. Discov. Med..

[44] Carrió I., Flotats A. (2020). Liquid Biopsies and Molecular Imaging: Friends or Foes?. Clin. Transl. Imaging.

[45] Ko J., Baldassano S.N., Loh P.-L., Kording K., Litt B., Issadore D. (2018). Machine Learning to Detect Signatures of Disease in Liquid Biopsies—A User’s Guide. Lab Chip.

[46] Xu J., Yang P., Xue S., Sharma B., Sanchez-Martin M., Wang F., Beaty K.A., Dehan E., Parikh B. (2019). Translating Cancer Genomics into Precision Medicine with Artificial Intelligence: Applications, Challenges and Future Perspectives. Hum. Genet..

[47] Shamseer L., Moher D., Clarke M., Ghersi D., Liberati A., Petticrew M., Shekelle P., Stewart L.A. (2015). Preferred Reporting Items for Systematic Review and Meta-Analysis Protocols (PRISMA-P) 2015: Elaboration and Explanation. BMJ.

[48] Whiting P.F., Rutjes A.W.S., Westwood M.E., Mallett S., Deeks J.J., Reitsma J.B., Leeflang M.M.G., Sterne J.A.C., Bossuyt P.M.M. (2011). QUADAS-2: A Revised Tool for the Quality Assessment of Diagnostic Accuracy Studies. Ann. Intern. Med..

[49] Borenstein M., Higgins J.P.T. (2013). Meta-Analysis and Subgroups. Prev. Sci..

[50] Deeks J.J., Macaskill P., Irwig L. (2005). The Performance of Tests of Publication Bias and Other Sample Size Effects in Systematic Reviews of Diagnostic Test Accuracy Was Assessed. J Clin. Epidemiol..

