# Supplementary material for: Cell-Free HPV-DNA as a Biomarker for Oropharyngeal Squamous Cell Carcinoma—A Step Towards Personalized Medicine?
Source: Cancers (Basel). 2020 Oct 15;12(10):2997. doi: 10.3390/cancers12102997 (PMC7602702; doi:10.3390/cancers12102997)
Supplement: Supplementary file 1 [file cancers-12-02997-s001.pdf]

Supplementary Materials: Cell-free HPV-DNA as Biomarker for Oropharyngeal Squamous Cell Carcinoma—A Step towards Personalized Medicine?

Nora Wuerdemann, Rishabh Jain, Anne Adams, Ernst-Jan M. Speel, Steffen Wagner, Simon A. Joosse and Jens P. Klussmann

Table S1. Extra information on studies included in the review and meta-analysis.

Study	Year	Target Gene	Volume	Treatment	HPV+ Tissue Status	Concentration of cfHPV-DNA (at first Diagnosis)	Tube	Follow up Time
Cao et al. [22]#**	2012	E6, E7, L1	NR	Chemoradiotherapy	p16 staining PCR/ HPV In-situ hybridization	Median = 222 copies/ml (0–5,500 copies/ml)	NR	12–22 months
Ahn et al. [23]#**	2014	E6, E7	NR	Surgery/Chemoradiotherapy	HPV In-situ hybridization qPCR	NR	Citrate	49 months
Dahlstrom et al. [24]#**	2015	E6/E7	500 µL	Chemoradiotherapy	PCR	<8000 copies/mL E6 Median = 83 copies/mL E7 Median = 43 copies/mL	NR	67 months
Wang et al. [25]#	2015	E7	6 mL	Surgery/Chemoradiotherapy	PCR	NR	NR	12 months
Kuhs et al. [26]#	2017	Antibody: HPV16- L1, E1, E2,E4, E6, E7 HPV6,11,18,31,33,45,58- E6	NR	Surgery/Chemoradiotherapy	PCR (DNA/RNA testing) p16 staining HPV In-situ hybridization	NR	NR	307 days
Lee et al. [27]#**	2017	39-amplicon single pool panel covering 34 distinct regions of HPV16 genome	5 mL	Chemoradiotherapy	p16 staining qPCR	NR	NR	12 weeks
Chera et al. [28]#	2019	E7	2–5 mL	Chemoradiotherapy	p16 staining	HPV16 *n* = 84 Median = 419 copies/mL(8–22,579 copies/mL) High risk HPV strain *n* = 8 Median = 124	BCT tubes (Streck)	16.5 months

						copies/mL (71–15,829 copies/mL)		
Damerla et al. [29]#	2019	E6,E7	4–5 mL	Surgery/Chemoradiotherapy	HPV In-situ hybridization p16 staining	Mean= 1,218 copies/mL (0 to 13,163 copies/mL)	BCT tubes (Streck) or BD vacutainer K2 EDTA tubes	NR
Nguyen et al. [30]#	2020	E7	NR	Chemoradiotherapy/ Immunotherapy	p16 staining	Median= 880 copies/mL (11–161,680 copies/mL)	EDTA	20 months
Reder et al. [31]#	2020	E6,E7	500 µL	Surgery/Chemoradiotherapy	p16 staining PCR	E6 Median= 9,283 copies/mL (0-79,850 copies/mL) E7 Median = 12,699 copies/ml (0-1,50,838 copies/ml)	BCT tubes (Streck)	NR
Mazurek et al. [32]	2016	E6,E7	1 mL	Chemoradiotherapy	NR	OPSCC 9.60 ± 6.23 ng/ml > other HNSCC 7.67 ± 4.44 ng/ml	NR	NR
Jeannot et al. [33]	2016	E7	200µL	NR	NR	4,605 copies/ml	NR	NR
Rutkowski et al. [34]	2017	E6,E7	1 mL	Chemoradiotherapy	NR	NR	K_3EDTA	Follow up time is given separately for each of the 55 patients
Hanna et al. [35]	2018	E7	NR	Cytotoxic/ Immunotherapy	p16 staining PCR/ HPV In-situ hybridization	Cutoff value = 5–10 copies/ml	NR	133 days
Veyer et al. [36]	2019	E6	200µL	NR	p16 staining/HPV16 molecular detection	Median = 319.4 copies/ml (0–1,47,416.7 copies/ml)	EDTA	2.84 years
Chera et al. [37]**	2020	E6, E7	2–5 mL	Chemoradiotherapy	p16 staining	NR	BCT tubes (Streck)	23 months

Abbreviations: NR- not reported, qPCR- Quantitative PCR, BCT- Blood collection tube, OPSCC- Oropharyngeal squamous cell carcinoma. # studies included in meta-analysis at first diagnosis and ** studies included in meta-analysis during follow-up.

Table S2. Quality assessment of the studies included in meta-analysis.

Study	Risk of Bias				Applicability Concerns		
	Patient Selection	Index Text	Reference Standard	Flow and Timing	Patient Selection	Index Text	Reference Standard
**Cao et al. [22]#**	L	L	L	L	L	L	L
**Ahn et al. [23]#**	L	L	L	L	L	L	L
**Dahlstrom et al. [24]#**	L	L	L	L	L	L	L
**Wang et al. [25]#**	L	L	L	L	L	L	L
**Kuhs et al. [26]#**	L	L	L	L	L	L	L
**Lee et al. [27]#**	L	L	L	L	L	L	L
**Chera et al. [28]#**	L	L	L	L	L	L	L
**Damerla et al. [29]#**	L	L	L	L	L	L	L
**Nguyen et al. [30]#**	H	L	L	L	H	L	L
**Reder et al. [31]#**	L	L	L	L	L	L	L

Abbreviations: L- Low risk of bias, H- High risk of bias. # studies included in meta-analysis at first diagnosis. # studies included in meta-analysis at first diagnosis.

Table S3. Primers and probes used in the different studies of the review and meta-analysis.

Studies	HPV-cfDNA Primers	Probes	Method
**Cao et al. [22]**#**	HPV16 E6/7<br>F- 5′-GAACCGAAACCGGTTAGTATAA-3′<br>R- 5′-ATGTATAGTTGTTTGCAGCTCTGT-3′<br>HPV18 E6/E7<br>F- 5′- GGACCGAAAACGGTGTATATAA-3′<br>R- 5′- CAGTGAAGTGTTCAGTTCGGT-3′<br>HPV L1<br>F- 5′- TTTGTTACTGTGGTAGATACATC-3′<br>R- 5′- GAAAAATAAACTGTAATCATATTC-3′	HPV16 E6/E7<br>5′-FAM-AGGACCCACAGGAGCGACCC-BHQ1 -3′<br>HPV18 E6/E7<br>5′-CO560-<br>ATGTGAGAAACACACCACAATACTATGGCGCG-BHQ1<br>-3′	qPCR
**Ahn et al. [23]**#**	HPV E6<br>F- 5′-TCAGGACCCACAGGAGCG-3′<br>R- 5′-CCTCACGTCGCAGTAACTGTTG-3′<br><br>HPV E7<br>F- 5′-CCGGACAGAGCCCATTACAA-3′<br>R- 5′-CGAATGTCTACGTGTGTGCTTTG-3′	HPV E6<br>5′-FAM-<br>CCCAGAAAGTTACCACAGTTATGCACAGAGCT-<br>TAMRA -3′<br><br>HPV E7<br>5′-FAM-CGCACAACCGAAGCGTAGAGTCACACT-<br>TAMRA -3′	qPCR
**Dahlstrom et al. [24]**#**	Not provided in the literature	Not provided in the literature	qPCR
**Wang et al. [25]**#	The primers used in this article was referenced to the study-Vogelstein, B., & Kinzler, K. W. (1999). Digital pcr. *Proceedings of the National Academy of Sciences*, *96*(16), 9236-9241.	The probes used in this article was referenced to the study-Vogelstein, B., & Kinzler, K. W. (1999). Digital pcr. *Proceedings of the National Academy of Sciences*, *96*(16), 9236-9241.	ddPCR
**Kuhs et al. [26]**#	-	-	Multiplex serologic testing
**Lee et al. [27]**#**	-	-	HPV16-detect
**Chera et al. [28]**#	HPV16<br>F- 5′- TGACTCTACGCTTCGGTTG -3′<br>R- 5′- GCCCATTAACAGGTCTTCC -3′<br><br>HPV18	HPV16<br>5′-FAM- CGTACAAAGCACACACGTAGACATTCGTAC–<br>ZEN -3′<br><br>HPV18	ddPCR

	F- 5′- TGAAGCCAGAATTGAGCTAG -3′<br>R- 5′- AGGACAGGGTGTTCAGAA -3′<br>HPV31<br>F- 5′- AGCACACAAGTAGATATTCGC -3′<br>R- 5′- TAGTAGAACAGTTGGGGCA -3′<br>HPV33<br>F- 5′- TAACACCACAGTTCGTTTATGT -3′<br>R- 5′- ACAATATTCACTGTGCCCATA -3′<br>HPV35<br>F- 5′- TGAGGCGACACTACGTC -3′<br>R- 5′- GTGCCCATTAATAAATCTTCCAA -3′	5′-LNA- CAGACGACCTTCG–HEX -3′<br>HPV31<br>5′-LNA- TAACAGCTCTTGC -HEX-3′<br>HPV33<br>5′-LNA- TGACCTACG AACC–HEX -3′<br>HPV35<br>5′-LNA- AGAGCACACACAT-HEX -3′	
**Damerla et al. [29]#**	HPV16<br>F- 5′- TATGCACAGAGCTGCAAACA -3′<br>R- 5′- GCAAAGTCATATACCTCACGTC -3′<br>HPV33<br>F- 5′- CCACAGTTCGTTTATGTGTCA -3′<br>R- 5′- TGCCCATAAGTAGTTGCTGT -3′	HPV16<br>5′-FAM- TGTGTGTACTGCAAGCAACAGTTACTG –lowaBlack -3′<br>HPV33<br>5′-FAM- AGTACAGCAAGTGACCTACGAACCA –lowaBlack -3′	ddPCR<br>qPCR
**Nguyen et al. [30]#**	Same as that of Jeannot et al.<br>HPV16<br>F- 5′- TCCAGCTGGACAAGCAGAAC -3′<br>R- 5′- CACAACCGAAGCGTAGAGTC -3′	Same as that of Jeannot et al.<br>HPV16<br>5′-FAM- ACAGAGCCCATTACAAT –Taqman probe -3′	ddPCR
**Reder et al. [31]#**	HPV E6<br>F- 5′- GCACCAAAAGAGAACTGCAATG -3′<br>R- 5′- GTTTGCAGCTCTGTGCATAACTG -3′<br>HPV E7<br>F- 5′- CAGCTCAGAGGAGGAGGATG -3′<br>R- 5′- GTAATGGGCTCTGTCCGGT -3′	HPV E6<br>5′-JOE- AGGACCCACAGGAGCGACCCAGAAAGTTAC-BHQ1 -3′<br>HPV E7<br>5′-FAM- AGATGGTCCAGCTGGACAAGCAG AACCGG-BHQ1 -3′	qPCR
**Mazurek et al. [32]**	HPV16<br>F- 5′- TTGCAGATCATCAAGAACACGTAGA -3′<br>R- 5′- CAGTAGAGATCAGTTGTCTCTGGTTGC -3′<br>HPV18<br>F- 5′- AGAGGCCAGTGCCATTCGT -3′<br>R- 5′- GTTTCTCTGCGTCGTTGGAGT -3′	HPV16<br>5′-FAM-AATCATGCATGGAGATACACCTACATTGCATGA-TAMRA -3′<br>HPV18<br>5′-FAM- TCCTGTCGTGCTCGGTTGCAGC -TAMRA-3′	TaqMan based TERT amplification

**Jeannot et al. [33]**	HPV16<br>F- 5′- TCCAGCTGGACAAGCAGAAC -3′<br>R- 5′- CACAACCGAAGCGTAGAGTC -3′<br>HPV18<br>F- 5′- AACATTTACCAGCCCGACGA -3′<br>R- 5′- TCGTCTGCTGAGCTTTCTAC -3′	HPV16<br>5′-FAM- ACAGAGCCCATTACAAT –Taqman probe -3′<br>HPV18<br>5′-FAM- AACCACAACGTCACACAA –Taqman probe -3′	ddPCR<br>qPCR
**Rutkowski et al. [34]**	Same as that of Mazurek et al.<br>HPV16<br>F- 5′- TTGCAGATCATCAAGAACACGTAGA -3′<br>R- 5′- CAGTAGAGATCAGTTGTCTCTGGTTGC -3′<br>HPV18<br>F- 5′- AGAGGCCAGTGCCATTCGT -3′<br>R- 5′- GTTTCTCTGCGTCGTTGGAGT -3′	Same as that of Mazurek et al.<br>HPV16<br>5′-FAM- AATCATGCATGGAGATACACCTACATTGCATGA- TAMRA -3′<br>HPV18<br>5′-FAM- TCCTGTCGTGCTCGGTTGCAGC -TAMRA-3′	TaqMan based TERT amplification
**Hanna et al. [35]**	HPV16<br>F- 5′- GCTCAGAGGAGGAGGATGAAATAGA -3′<br>R- 5′- GAGTCACACTTGCAACAAAAGGTT -3′<br>HPV18<br>F- 5′- AGGAAGAAAACGATGAAATAGATGGAGTT -3′<br>R- 5′- GGCTTCACACTTACAACACATAC -3′<br>HPV31<br>F- 5′- CAGCTCAGATGAGGAGGATGTC -3′<br>R- 5′- ACTTACACTGACAACAAAAGGTAACGA -3′<br>HPV33<br>F- 5′- CGGCCAGATGGACAAGCA -3′<br>R- 5′- TGCTGTACTGTTGACACATAAACGA -3′<br>HPV45<br>F- 5′- CAGATGGAGTTAGTCATGCACAAC -3′<br>R- 5′- CCGTCACACTTACAACATACACACA -3′	HPV16<br>5′-FAM- CCGGACAGAGCCCATTACAAT –NFQ -3′<br>HPV18<br>5′-FAM- ACCAGCCCGACGAGCCGA –NFQ -3′<br>HPV31<br>5′-FAM- AACCGGACACATCCAATTAC -NFQ-3′<br>HPV33<br>5′-FAM- ACCAGCCACAGCTGATTAC –NFQ -3′<br>HPV45<br>5′-FAM- CCGACGAGCCGAACCACAG -NFQ -3′	ddPCR
**Veyer et al. [36]**	Not provided in the literature	Not provided in the literature	ddPCR
**Chera et al. [37]** **	Same as that of Chera et al.<br>HPV16	Same as that of Chera et al.<br>HPV16	ddPCR

F- 5′- TGACTCTACGCTTCGGTTG -3′	5′-FAM- CGTACAAAGCACACACGTAGACATTCGTAC–
R- 5′- GCCCATTAACAGGTCTTCC -3′	ZEN -3′
HPV18	HPV18
F- 5′- TGAAGCCAGAATTGAGCTAG -3′	5′-LNA- CAGACGACCTTCG–HEX -3′
R- 5′- AGGACAGGGTGTTCAGAA -3′	
	HPV31
HPV31	5′-LNA- TAACAGCTCTTGC -HEX-3′
F- 5′- AGCACACAAGTAGATATTCGC -3′	
R- 5′- TAGTAGAACAGTTGGGGCA -3′	HPV33
	5′-LNA- TGACCTACG AACC–HEX -3′
HPV33	
F- 5′- TAACACCACAGTTCGTTTATGT -3′	HPV35
R- 5′- ACAATATTCACTGTGCCCATA -3′	5′-LNA- AGAGCACACACAT-HEX -3′
HPV35	
F- 5′- TGAGGCGACACTACGTC -3′	
R- 5′- GTGCCCATTAATAAATCTTCCAA -3′	

Abbreviations: NR- not reported, qPCR- Quantitative PCR, ddPCR- Droplet digital PCR. # studies included in meta-analysis at first diagnosis and ** studies included in meta-analysis during follow-up.

© 2020 by the authors. Licensee MDPI, Basel, Switzerland. This article is an open access article distributed under the terms and conditions of the Creative Commons Attribution (CC BY) license (http://creativecommons.org/licenses/by/4.0/).
